# Supplementary material for: Vaccination and variants: Retrospective model for the evolution of Covid-19 in Italy
Source: PLoS One. 2022 Jul 8;17(7):e0265159. doi: 10.1371/journal.pone.0265159 (PMC9269459; doi:10.1371/journal.pone.0265159)
Supplement: S1 Fig — (PDF) [file pone.0265159.s001.pdf]

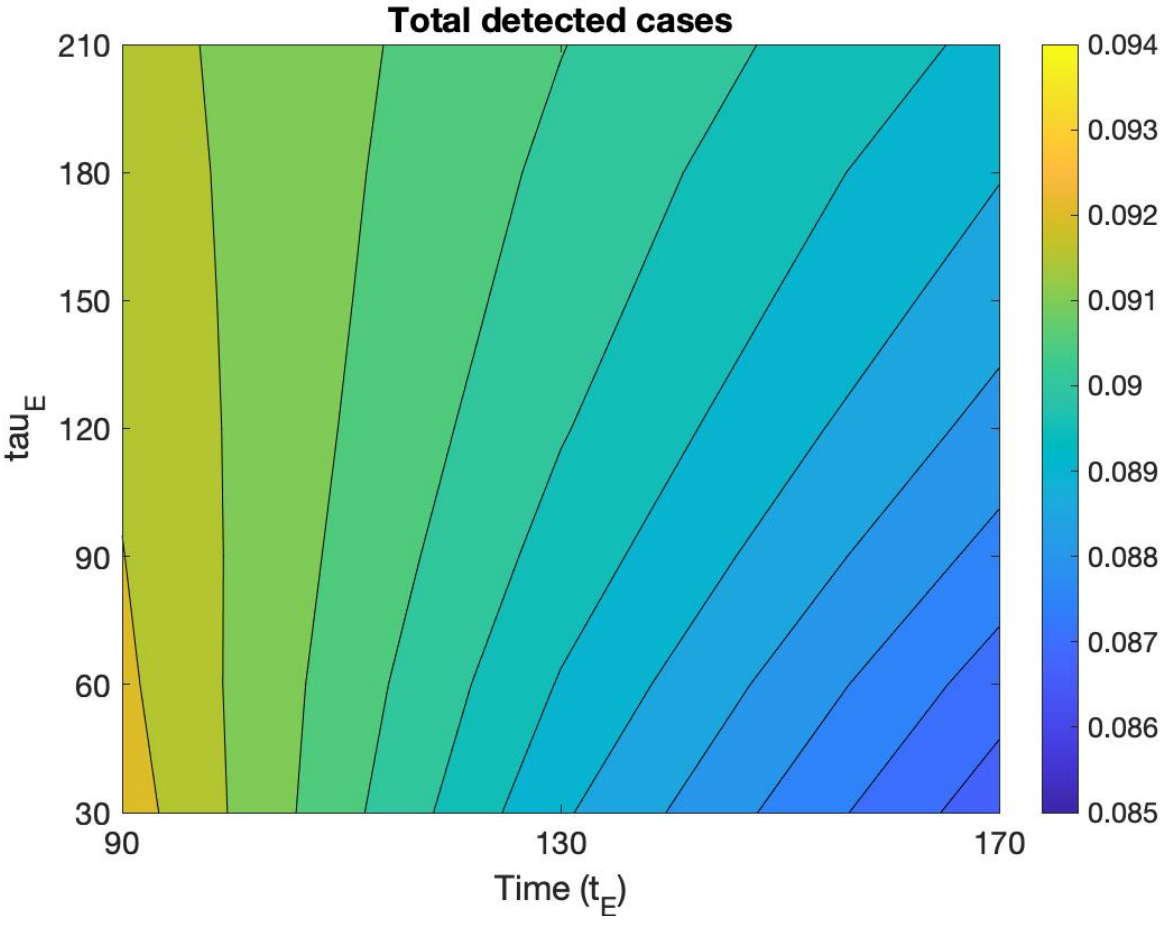

S1 Fig: Total number of detected cases at the final day of simulation (December 16, 2021) as a function of the decay interval τ_E and the medium time of antibody decay, t_E.
